# Screening for mouse genes lost in mammals with long lifespans

**DOI:** 10.1186/s13040-019-0208-x

**Published:** 2019-11-09

**Authors:** Lev I. Rubanov, Andrey G. Zaraisky, Gregory A. Shilovsky, Alexandr V. Seliverstov, Oleg A. Zverkov, Vassily A. Lyubetsky

**Affiliations:** 1 Institute for Information Transmission Problems of the Russian Academy of Sciences (Kharkevich Institute) IITP RAS, 19 build. 1 Bolshoy Karetny per., Moscow, 127051 Russia; 2 Shemyakin-Ovchinnikov Institute of Bioorganic Chemistry, Russian Academy of Sciences (IBCH RAS) 16/10, Miklukho-Maklaya str., Moscow, 117997 Russia

**Keywords:** Lifespan, Longevity, Aging, Gerontology, Gene loss, Synteny, In silico analysis

## Abstract

**Background:**

Gerontogenes include those that modulate life expectancy in various species and may be the actual longevity genes. We believe that a long (relative to body weight) lifespan in individual rodent and primate species can be due, among other things, to the loss of particular genes that are present in short-lived species of the same orders. These genes can also explain the widely different rates of aging among diverse species as well as why similarly sized rodents or primates sometimes have anomalous life expectancies (e.g., naked mole-rats and humans). Here, we consider the gene loss in the context of the prediction of Williams’ theory that concerns the reallocation of physiological resources of an organism between active reproduction (r-strategy) and self-maintenance (K-strategy). We have identified such lost genes using an original computer-aided approach; the software considers the loss of a gene as disruptions in gene orthology, local gene synteny or both.

**Results:**

A method and software identifying the genes that are absent from a predefined *set* of species but present in another predefined *set* of species are suggested. Examples of such pairs of sets include long-lived vs short-lived, homeothermic vs poikilothermic, amniotic vs anamniotic, aquatic vs terrestrial, and neotenic vs nonneotenic species, among others. Species are included in one of two sets according to the property of interest, such as longevity or homeothermy. The program is universal towards these pairs, i.e., towards the underlying property, although the sets should include species with quality genome assemblies. Here, the proposed method was applied to study the longevity of Euarchontoglires species. It largely predicted genes that are highly expressed in the testis, epididymis, uterus, mammary glands, and the vomeronasal and other reproduction-related organs. This agrees with Williams’ theory that hypothesizes a species transition from r-strategy to K-strategy. For instance, the method predicts the mouse gene *Smpd5*, which has an expression level 20 times greater in the testis than in organs unrelated to reproduction as experimentally demonstrated elsewhere. At the same time, its paralog *Smpd3* is not predicted by the program and is widely expressed in many organs not specifically related to reproduction.

**Conclusions:**

The method and program, which were applied here to screen for gene losses that can accompany increased lifespan, were also applied to study reduced regenerative capacity and development of the telencephalon, neoteny, etc. Some of these results have been carefully tested experimentally. Therefore, we assume that the method is widely applicable.

## Background

The genetic propensity of certain species for longevity and anti-aging is a challenging problem in vertebrate biology. Of particular interest are the genes that influence life expectancy differences among species. These genes are expected to be the real longevity genes of interest and should explain the wide differences in the rate of aging among diverse species and why similarly sized rodents or primates such as naked mole-rats (NMR) or humans sometimes have anomalous life expectancies (see, e.g., [1]). According to [2], no such genes have been unequivocally identified. We performed a computer-aided analysis of data relevant to lifespan and made a bioinformatic search for the genes, the loss of which might modulate lifespan. This search is based on the general idea that such genes are lost in a predefined *set* of species but are present in another predefined *set* of species. Examples of such pairs of sets include long-lived vs short-lived, homeothermic vs poikilothermic, amniotic vs anamniotic, aquatic vs terrestrial, and neotenic vs nonneotenic species, among others. Species are included in one of two sets depending on the property of interest, such as longevity or homeothermy. A bioinformatics method and software relevant to the idea are universal towards these sets and the property that defines them. Earlier, we used the method to predict that the loss of certain genes in the evolution of vertebrates could increase the size of the telencephalon in hot-blooded vertebrates relative to cold-blooded vertebrates at the expense of a considerably decreased regenerative capacity, and later it was experimentally demonstrated [3]. Accordingly, we considered homeotherms vs poikilotherms among vertebrates there. Here, we studied Euarchontoglires in regard to their lifespan with due account to the body weight [4, 5]. The main concepts of the approach have been implemented in the lossgainRSL software [6]. The results of this work were reported at the conference [7].

**Fig. 1:**
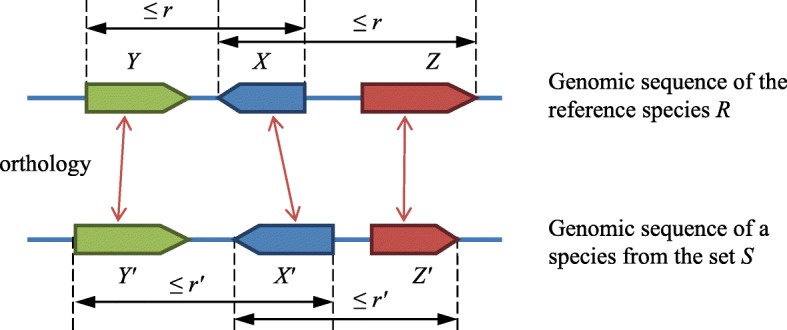
The elementary condition of the presence of a gene *X* from *R* in another species from the set *S* considering the synteny. The values of *r* and *r’* are fixed for the pair (*R*, *S*)

The well-known sentence “less is more” can be interpreted as the significance of screening for lost genes, which nevertheless lead to largely positive evolutionary events for the species (e.g., [8]). Generally, gene loss is considered a combination of changes in the gene nucleotide composition, exon-intron structure, tissue-specific expression, and gene function, as well as of significant changes in its local synteny. However, the computer-aided evaluation of the components of this notion is complicated, which is particularly evident in regard to gene expression or function. We interpret the local synteny as the orthology of genes *X* and *X’* in two different species that is accompanied by orthologous pairs of other genes, *Y* and *Y′*, *Z* and *Z’*, etc., located in the vicinity of *X* and *X’*, respectively, as shown in Fig. 1. This definition depends on a number of parameters that are detailed in the Methods section. These include the numerical parameters such as sizes *r* and *r*’ of the vicinities, and the tabular parameters such as the gene orthology relation inferred by the reconciliation of the gene trees with the species tree (with no account for synteny) as presented in the Ensembl Compara database [9]. Note that original orthology inference methods [3] can be used as an alternative to or in combination with the method used in Ensembl Compara as well as the method based on the reconstruction of chromosome structures proposed in [10, 11]. The problem of orthology inference is quite complicated and far from being completely solved. Different methods do not always yield consistent orthology predictions even for evolutionarily close mammals such as mice and rats. The approaches to this problem have been reviewed, e.g., in [12]. The solutions obtained by popular algorithms have been compared in [13].

Thus, the goal of this paper was to describe a method and software for lost gene identification and to illustrate their application for studying the longevity of Euarchontoglires species. The program identifies the genes that are present in a predefined set of species but absent from another predefined set of species. Here, these sets are defined by the longevity property of Euarchontoglires. The program is universal in regard to its applicability to any property, although the corresponding sets should include species with quality genome assemblies, which do not always match the best biological choice. The program has not been described previously and implements the very part of our method that is required for the current property of longevity. Thus, we have obtained a list of mouse genes lost by long-lived Euarchontoglires, which can be linked to their longevity. Supraprimates were chosen since this is the natural clade combining primates and rodents with a wide variance in lifespans. In addition, this taxon is well represented in databases. Further data on the considered species and individual examples of lost genes are given in Additional file 1.

If the complex set of conditions is tested against a multitude of species, data mining reveals patterns that are hardly discernible when few genomes are analyzed. Data mining raises the requirements for computation performance and software flexibility. Here, this is achieved by supercomputer-based parallel processing and the separation of the system of conditions from the programming code, which is realized by describing the gene selection procedure in the specifically designed language. This language allows the easy variation of the conditions. The prediction accuracy is improved by the combined application of several orthology inference methods and variation of all significant parameters, which also increases the computational load.

## Results

### The method of in silico search for lost genes

This section presents an original approach to the identification of lost (or, similarly, acquired) genes, which is the major achievement of this study; its computer implementation (with technical details relevant to the corresponding software) can be found in the Methods section. We are given *d* groups of species (each group is a taxon or a union of taxa) and a species-specific property *T*. Two *fractions* of each group are distinguished by the property, the *upper* fraction where the property is present and the *lower* one where it is missing. The species that belong to the upper and lower fractions will be referred to as *upper* and *lower*, respectively. Let us specify one of the lower species as the *reference* species, assuming that its genome is well assembled and convenient for further wet experiments. The genes of the reference species present in the genomes of the lower species and missing (lost) in the genomes of the upper species are identified in silico. For example, *T* can indicate longevity or homeothermy, etc. It is unlikely that *each* of the identified genes is directly linked to a corresponding property that is inherent in the respective upper species, due to incomplete genomic assemblies as well as to the complexity of the evolution process. However, we believe that the property *T* that defines the set of upper species was formed in evolution, in particular, due to the loss of some genes from those predicted. In other words, such a link with the property *T* can be observed for some of the identified genes by the proposed approach. Our approach and software can be of general interest.

**Fig. 2:**
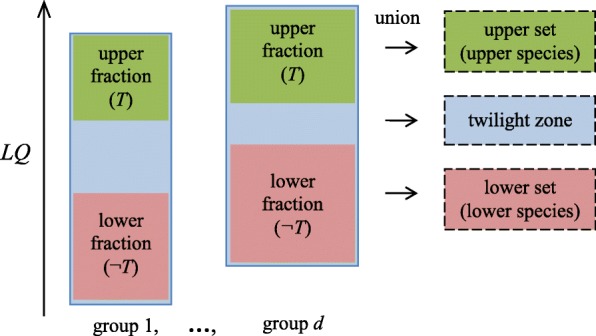
Upper and lower fractions that were formed by *Т* using the boundaries for the longevity quotient *LQ*; the boundaries depend on a given group

Here, *Т* is longevity; accordingly, the upper and lower fractions are distinguished by the longevity quotient (*LQ*) boundaries (Fig. 2).

**Fig. 3:**
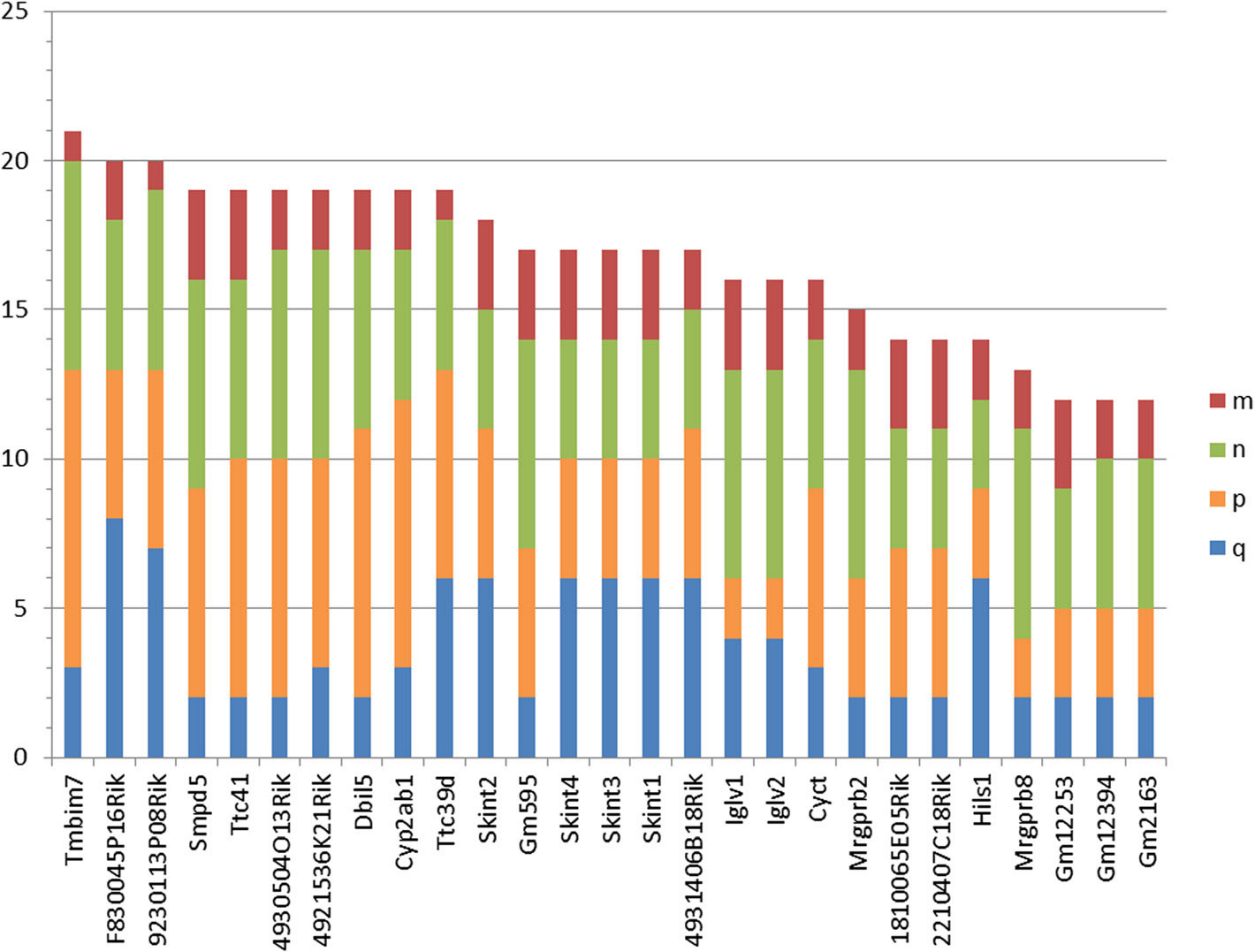
The quality index of the prediction of lost genes. Abscissa, program-predicted lost genes; ordinate, the value of *S=m + n + p+ q*. For each gene, *m* and *n* are the number of upper glires and primates, respectively, in which the gene was lost, and *p* and *q* are the number of lower glires and primates, respectively, in which the gene is present

It is natural to refine the problem to identify the genes lost not in all of the upper species and represented not in all of the lower species but rather those lost in *at least m*, *n*, ... upper species and represented in *at least p*, *q*, ... lower species (group parameters). In addition, the upper and lower species can be chosen such that the absence or presence of the sought-for gene is compulsory. These parameters improve the prediction reliability for evolutionarily distant species and imperfect assemblies. Note that the sum *S= m + n...p+q*....can be used as a quality index of the prediction (Fig. 3).

The absence or presence of a gene from the reference species in the genomes of other species is considered without accounting for orthology type: 1-to-1, 1-to-many, or many-to-many. Moreover, different orthologу inference methods can produce different results. Therefore, our method of lost gene prediction combines the following steps to identify gene presence.

(A)  Several orthology inference methods are independently used, and the gene “presence” or “absence” is confirmed by the presence or absence, respectively, of local synteny (see the Methods section for details).
(B) The decision of gene presence or loss is made by consensus or voting for the results of (А).
(C) If the absence of an ortholog favors the loss of gene *X* in the reference species, but the genomic alignment contains a gene *X’* in the tested species such that the exon/intron structures of *X* and *X’* have significant similarity, including a satisfactory local alignment of certain exons, *X’* is considered as substantially varied version of gene *X*.
(D) In the above case, similar tissue expression patterns of genes *X* and *X’* or their similar biochemical roles serve as an indication of the presence of gene *X* in the tested species. For instance, the predominant expression of both genes in the same organs favors the presence of the gene in the tested species.

Naturally, the result depends on the parameters. For each pair of species, the choice that should be made is the number of orthologous gene pairs in the vicinity that is sufficient to acknowledge the presence of the gene (in Fig. 1, this number equals 2). Each vicinity is described by a size *r*, which depends on the upper or lower fraction as well as on the group of species. Here, the gene is finally considered lost if it was lost within *r’* running from some lower boundary to the upper boundary *r*_0_. The reason for choosing *r*_0_ is based on the following considerations. The 3D structure of DNA includes topologically associated domains (TADs); the impact of enhancers is usually limited to the TAD size so that transcription control largely occurs within the TAD. Local synteny conservation is usually considered as its conservation within the same TAD. In mammals, the TAD size ranges from one to several Mbp [14–17]. While choosing *r*_0_ can be problematic in general, it can be justified here.

Our fast and flexible program lossgainRSL [6] identifies lost and acquired genes between several groups of species. Currently, it implements step (A) separately for each orthology inference method, while steps (В), (C), and (D) are performed during computer-aided postprocessing. Their validation can be automated as well, but this was not implemented in the current program since the automatic validation is less accurate and requires its own thresholds that are hard to clearly define. The details of this implementation can be found in the Methods section.

### Mouse genes lost in long-lived Euarchontoglires

Here, longevity was considered as the abovementioned property *T*. Calculations were performed for many variants, one of which is presented here. Specifically, we studied the set of 40 Euarchontoglires species listed in Table 1 and divided them into two groups (*d* = 2), the first consisting of 16 rodents and lagomorphs; and the second consisting of 24 primates. The upper set included 10 species (3 glires and 7 primates); the lower, 21 species (11 glires and 10 primates) and the mouse was used as the reference species. The compulsory upper species were the naked mole-rat, human, and white-headed capuchin; the compulsory lower species was the mouse. The following parameters were used: *m* = 1, *n* = 2, *p* = 2, and *q* = 2; the arguments for using these values are presented in the Methods. We are not sure that these values are the best possible considering that the lifespan (longevity) combines many complex phenomena including resistance to cancer [21]. We do not pretend to analyze longevity itself, which is a separate and difficult biological task, but mainly present a fast and freely available program as well as exemplify its application.

**Table 1:** Species of rodents, lagomorphs, and primates. Maximum reported lifespan (MRLS, years) and longevity quotient (LQ,%) were obtained from the AnAge database [18]

Species	Common name	MRLS	LQ
*Heterocephalus glaber*	Naked mole-rat (NMR)	31	368
*Nannospalax galili* (*Spalax galili*)	Upper Galilee mountains blind mole rat (BMR)	20.2	190
*Fukomys damarensis* (*Cryptomys damarensis*)	Damaraland mole-rat (DMR)	15.5	143
*Chinchilla lanigera*	Long-tailed chinchilla	17.2	131
*Octodon degus*	Common degu	14	124
*Dipodomys ordii*	Kangaroo rat	9.9	109
*Peromyscus maniculatus bairdii*	Northern American deer mouse	8.3	107
*Cavia porcellus*	Guinea pig	12	89
*Jaculus jaculus*	Lesser Egyptian jerboa	7.3	81
*Ictidomys tridecemlineatus* (*Spermophilus tridecemlineatus*)	Thirteen-lined ground squirrel	7.9	73
*Microtus ochrogaster*	Prairie vole	5.3	61
*Oryctolagus cuniculus* (*Lepus cuniculus*)	European rabbit	9	58
*Mus musculus*	Mouse	4	51
*Cavia aperea*	Brazilian guinea pig	6	50
*Mesocricetus auratus*	Golden hamster	3.9	39
*Rattus norvegicus*	Common rat	3.8	32
*Homo sapiens*	Human	122.5	463
*Cebus capucinus imitator*	White-headed capuchin	54.8	331
*Nomascus leucogenys*	Northern white-cheeked gibbon	44.1	238
*Pan troglodytes*	Chimpanzee	59.4	236
*Saimiri boliviensis*	Bolivian squirrel monkey	30.3	232
*Pan paniscus*	Bonobo	55	222
*Pongo abelii*	Sumatran orangutan	59	221
*Aotus nancymaae*	Nancy Ma’s night monkey	30^**a**^	218^**a**^
*Macaca fascicularis*	Crab-eating macaque	39	209
*Macaca mulatta*	Macaque	40	206
*Gorilla gorilla gorilla*	Western lowland gorilla	60.1	200
*Callithrix jacchus*	Marmoset	22.8	200
*Microcebus murinus*	Mouse lemur	18.2	197
*Macaca nemestrina*	Pigtail macaque	37.6	194
*Colobus angolensis palliatus*	Angola colobus	35.3	180
*Mandrillus leucophaeus*	Drill	39	177
*Chlorocebus sabaeus*	Green monkey	31.6	173^**b**^
*Propithecus coquereli*	Coquerel’s sifaka	30.5	170^**c**^
*Carlito syrichta*	Philippine tarsier	16	157
*Rhinopithecus roxellana*	Golden snub-nosed monkey	29.5	139
*Otolemur garnettii*	Bushbaby	20	136
*Cercocebus atys*	Sooty mangabey	26.8	135^**d**^
*Papio anubis*	Olive baboon	25.2	114^**e**^
*Rhinopithecus bieti*	Black snub-nosed monkey	15	75^f^

^**a**^The lifespan is not known; however, it is 33.8, 30.1 [19], and 30 [20] years in related night monkeys *Aotus lemurinus*, *A. trivirgatus*, and *A. azarae*, respectively. LQ was calculated for a body weight of 874 g, which is the median weight for these species

^**b**^For a body weight of 5.62 kg as specified in AnAge

^**c**^For a body weight of 5 kg, which is the median weight for related species *Propithecus verreauxi*, *P. diadema*, and *P. tattersalli*

^**d**^For a body weight of 9.39 kg, which is the median weight for related species *Cercocebus torquatus*, *C. galeritus*, *C. agilis*, and *Lophocebus albigena*

^**e**^For the mean male/female body weight

^**f**^For a body weight of 9.96 kg as specified in AnAge

For the sets and parameters specified and the Ensembl v95 orthologs, the program identified 134 lost mouse genes listed in Additional file 2: Table S1. The result of postprocessing is shown in the second column, where zero indicates that the corresponding gene is presented as a result of steps (C)-(D). After that, 70 genes remain, which are discussed below and in Additional file 3. These 70 genes include 43 olfactory receptor genes, many of which are expressed in the vomeronasal organ, as well as 27 more genes, which are provisionally called meaningful and shown in Table 2. In this calculation, step (B) was not applied since the orthologs and paralogs were retrieved from the Ensembl v95 database.

**Table 2:** Meaningful mouse genes lost in long-lived mammals

Ensembl ID	Chr.	Gene symbol	Description	Max. expression	
ENSMUSG00000013353	7	*4931406B18Rik*	RIKEN cDNA 4931406B18 gene	testis	*P*
ENSMUSG00000013653	11	*1810065E05Rik*	RIKEN cDNA 1810065E05 gene	stomach, caecum	*–*
ENSMUSG00000014529	5	*Tmbim7*	transmembrane BAX inhibitor motif containing 7	testis	*P*
ENSMUSG00000020434	11	*4921536K21Rik*	RIKEN cDNA 4,921,536 K21 gene	testis	*F*
ENSMUSG00000022818	16	*Cyp2ab1*	cytochrome P450, family 2, subfamily ab, polypeptide 1	heart, spleen, placenta	*P*
ENSMUSG00000034359	4	*Skint2*	selection and upkeep of intraepithelial T cells 2	thymus, skin	*–*
ENSMUSG00000037145	11	*2210407C18Rik*	RIKEN cDNA 2210407C18 gene	stomach	*–*
ENSMUSG00000038057	11	*Dbil5*	diazepam binding inhibitor-like 5	testis	*P*
ENSMUSG00000038994	11	*Hils1*	histone H1-like protein in spermatids 1	testis	*P*
ENSMUSG00000043727	2	*F830045P16Rik*	RIKEN cDNA F830045P16 gene	spleen, MPC	*P*
ENSMUSG00000044937	10	*Ttc41*	tetratricopeptide repeat domain 41	testis	*P*
ENSMUSG00000046196	17	*Ttc39d*	tetratricopeptide repeat domain 39D	testis	*P*
ENSMUSG00000050425	7	*Mrgprb2*	MAS-related GPR, member B2	skin, ovary, forelimb	*–*
ENSMUSG00000050870	7	*Mrgprb8*	MAS-related GPR, member B8	testis	*–*
ENSMUSG00000052642	11	*4930504O13Rik*	RIKEN cDNA 4930504O13 gene	testis	*P*
ENSMUSG00000055960	4	*Skint4*	selection and upkeep of intraepithelial T cells 4	skin, testis, thymus, brain	*–*
ENSMUSG00000056436	2	*Cyct*	cytochrome c, testis	testis	*P*
ENSMUSG00000058287	11	*Gm12253*	predicted gene 12,253	many organs	*–*
ENSMUSG00000070868	4	*Skint3*	selection and upkeep of intraepithelial T cells 3	skin, spleen, lung	*–*
ENSMUSG00000071724	15	*Smpd5*	sphingomyelin phosphodiesterase 5	testis	*P*
ENSMUSG00000076934	16	*Iglv1*	immunoglobulin lambda variable 1	spleen, colon, thymus	*P*
ENSMUSG00000076940	16	*Iglv2*	immunoglobulin lambda variable 2	spleen, thymus, colon	*P*
ENSMUSG00000078722	4	*Gm12394*	predicted gene 12,394	brain, testis	*F*
ENSMUSG00000078934	9	*9230113P08Rik*	RIKEN cDNA 9230113P08 gene	MPC, brain	*F*
ENSMUSG00000079606	X	*Gm595*	predicted gene 595	testis	*P*
ENSMUSG00000089773	4	*Skint1*	selection and upkeep of intraepithelial T cells 1	skin, whole organism, thymus	*P*
ENSMUSG00000095779	4	*Gm2163*	predicted gene 2163	brain, testis	*F*

Columns, left to right: Ensembl gene ID, chromosome, gene symbol, gene/protein description, and the organ(s) with the highest gene expression(s). The rightmost column employs the following designations: *P*, the mouse gene has orthologous flanking genes in humans but is specified as a pseudogene in Ensembl; *F*, the mouse gene has orthologous flanking genes in humans but is not specified in Ensembl; ‘–’, no alignment of the corresponding regions is available in Ensembl. MPC, medial nasal prominence

A considerable part of the predicted genes are still detected as lost when the algorithm parameters are varied. For instance, only 3 out of 27 genes in Table 2 are missing at *m* = 2: *Tmbim7*, *9230113P08Rik*, and *Ttc39d*; similarly, three genes are missing at *p* = 3: *Iglv1*, *Iglv2*, and *Mrgprb8*; and a single gene, *Hils1,* is missing at *n* = 3. Many of the genes in Table 2 were predicted irrespective of the orthology inference method. Complete data related to this special problem are not presented here. However, we compared the screening results for the abovementioned 40 Euarchontoglires species produced by three alternative orthology inference methods: Ensembl v92 employing a distinct approach, our original method of protein clustering [22], and blastp identification of reciprocal best hits with 0.001 cutoff including different voting schemes. These alternative methods predict at least genes most reasonably linked to longevity. Specifically, using the “2 of 3” voting scheme identified, apart from the olfactory receptors, 13 genes: *Tmbim7*, *4921536K21Rik*, *Cyp2ab1*, *Skint2*, *Dbil5*, *Hils1*, *Ttc41*, *Ttc39d*, *4930504O13Rik*, *Cyct*, *Gm12253*, *Smpd5*, and *9230113P08Rik*. The consensus of the three orthology inference methods excludes the three underlined genes. A comparison of numerous thusly generated lists of lost genes is hardly practicable since it is hard to interpret genes that are present in one list and absent from another. The elucidation of the role of a lost gene depends on a component of longevity under consideration and requires analysis of individual gene properties.

## Discussion

There are numerous theories of aging based on diverse concepts of this extremely complex process. These include Williams’ antagonistic pleiotropy theory [23] relating the active reproduction vs longevity opposition, thus opposing the r- and K-strategies for the maintenance of the physiological functions of the body. Indeed, the predicted lost genes largely demonstrate a specific relationship to reproductive-related organs, which agrees with Williams’ hypothesis [23] concerning the reallocation of the physiological resources of the body between self**-**maintenance and reproduction (transition to К-strategy in the species evolution). We are unaware of software for large-scale screening for the genes, the loss of which correlates with species-specific lifespans in the context of Williams’ theory [23]. Published data indicate that many genes are actively expressed in mouse testes and correspond to human pseudogenes, although such genes were identified with no account for synteny [24]. We searched mouse genes whose orthology and local genomic synteny were present in short-lived species, but either of these relations was disrupted in long-lived species. Our program can simultaneously process a vast set of available species and all their known genes. Many of the genes identified by the program are essential for reproduction. Accordingly, their loss can indicate the transition to Williams’ K-strategy in the species evolution [23].

The global problem here is whether gene loss can cause a significant evolutionary change. We also asked this question until our extensive three-year experiments demonstrated that the tropical clawed frog’s gene, ENSXETG00000033176, identified by the same program was, to say the least, associated with a sharp drop in regenerative potential as well as with forebrain development [3].

The list of 134 lost genes in Additional file 2 was obtained for the following long-lived species: 3 rodents with LQ > 130% and 7 primates with LQ > 220%, assuming that the genes should necessarily be lost in the NMR, human and white-headed capuchin. The lower set included 11 rodents and lagomorphs with LQ < 110% and 10 primates with LQ < 190%. The mouse was the reference species. We validated these genes with an account of the genomic and nucleotide alignments, exon-intron structure, the similarity of organ-specific expression, and gene function using well-known bioinformatics tools such as Ensembl Genome Browser [25] and Expression Atlas [26]. This was done for pairs of genes suspected as orthologs in mouse and a long-lived species that were not specified as orthologs or paralogs in Ensembl. For instance, synteny conservation can be evident from the genomic alignments and thus favor the orthology of the gene pair, although the genes are not considered orthologous. Approximately half of these 134 genes lacked such signs of orthology and synteny, which we considered validating the prediction quality. These genes include 43 olfactory genes and 27 other genes that were provisionally designated as the meaningful. More than one-third of olfactory genes are significantly (data not shown) expressed in the testis and vomeronasal organ, both of which are associated with reproduction. Similarly, the bulk of the meaningful genes demonstrate significant expression in organs associated with reproduction. These genes encode regulatory factors or enzymes linked to biochemical pathways critical for reproduction; this particular statement is not discussed further here. The loss of some predicted vomeronasal and olfactory receptor genes in human and naked mole-rat conforms to their specific anatomical features. We consider these arguments to be relevant to the testing of our program on biological data.

Let us consider some of the 27 predicted meaningful genes. Certain mouse genes were lost completely (i.e., have no orthologs) or became pseudogenes in all long-lived species; however, their close paralogs were preserved in these species. For example, the *Smpd5* gene (ENSMUSG00000071724) encodes neutral sphingomyelinase 5; it localizes to both mitochondria and the endoplasmic reticulum. Its expression in the adult testis is approximately 20 times higher than in any other organ. According to Mouse ENCODE [27], its expression in adult testis is 60 RPKM, while the expression in other organs does not exceed 3 RPKM. According to the Expression Atlas [26], the *Smpd5* expression level in the testis varied from 110 to 185 TPM in six experiments but never exceeded 8 TPM in any other tissue. Its paralog *Smpd3* (ENSMUSG00000031906), which encodes another neutral sphingomyelinase, is actively expressed in many mouse organs, including the brain, intestine, and testis. It does not stand out in terms of expression in reproduction-related organs and is not identified by our program. Sphingomyelinases hydrolyze the membrane phospholipid sphingomyelin into phosphocholine and ceramide, a component of transmembrane pores [28, 29]; *Smpd3* is likely involved in the release of neuromediators in the synaptic cleft [30]. In addition, sphingomyelinases can trigger apoptosis through the formation of ceramide pores in the mitochondrial membrane [31–33] and phospholipid transformation [34]. Sphingomyelin participates in the formation of the intracellular mitochondrial network in skeletal muscle, which is significantly decelerated in NMR relative to the mouse. Hence, *Smpd5* loss can serve as a prerequisite for neoteny. This and other human and NMR neotenic properties have been summarized and discussed in detail by Skulachev et al. [35, 36]. The relationship between the lifespan and certain sphingomyelinases in different animal species has been considered in [37–39].

The mouse gene *Dbil5* (ENSMUSG00000038057), which encodes diazepam binding inhibitor-like 5, is massively expressed in the testis (a 100-fold expression difference is shown in six experiments in [26] compared to other organs). This protein, located in the cytoplasm, binds fatty-acyl-CoA and mediates spermatogenesis [40]. In humans, it corresponds to the pseudogene *DBIL5P*; the neighboring genes were also preserved. In the Damaraland mole-rat (DMR, *Fukomys damarensis*), the neighboring genes were preserved, but there is no ortholog of mouse *Dbil5*. Genes orthologous to *Dbil5* are found in the African bush elephant, most Laurasiatheria species including the little brown bat, the rabbit, most rodents including the BMR, and many primates including the bushbaby, mouse lemur, Coquerel’s sifaka, macaque, and gibbon. The alignment of amino acid sequences suggests that the proteins in the degu, chinchilla, macaque, and gibbon substantially differ from those in the bushbaby, mouse lemur, Coquerel’s sifaka, rabbit, and some rodents. In other words, the proteins in the lemurs (bushbaby and mouse lemur) and some rodents form a cluster of similar proteins, while these proteins in the degu, chinchilla, macaque, and gibbon substantially differ from those in the cluster as well as from each other. Thus, a second protein cluster is not formed.

The mouse gene *Tmbim7* (ENSMUSG00000014529) encodes a transmembrane protein, a putative apoptosis inhibitor; it is actively expressed in the mouse testis (a 100-fold difference in eight experiments in [26] compared to other organs) and corresponds to the human pseudogene *TMBIM7P*. The gene corresponds to the pseudogene ENSGL00000028944 in the female NMR; however, the corresponding gene was preserved in the DMR. Without regard to synteny, the gene aligns with the gene *TMBIM6*, which is overexpressed in certain cancer types and suppresses Bax-induced apoptosis.

The loss of the flehmen response and olfactory receptors (abundantly represented in Additional file 2) in primates relative to other mammals is thought to be associated with reproduction but also with the acquisition of trichromatic vision [41].

The following two genes are identified by the program using different parameter *q* values than those considered above, *q* = 1 and *q* = 0, respectively.

The mouse gene *Wap* (ENSMUSG00000000381) has been lost completely or has become a pseudogene in the NMR, DMR, human, bonobo, chimpanzee, western gorilla, white-cheeked gibbon, and white-headed capuchin. It has an ortholog in a single short-lived primate (vervet monkey) and exists in the Bolivian squirrel monkey. The *Wap* gene encodes the whey acidic protein (WAP). WAP was described in bovine colostrum [42], its minor quantities are detectable in mouse milk [43], and it constitutes a considerable fraction of the total protein in rabbit milk [44, 45]. This protein inhibits *Staphylococcus aureus* growth and regulates the proliferation of mammary epithelial cells by preventing serine protease from degrading laminin, thus suppressing the MAP kinase pathway [46, 47]. WAP expression is upregulated during lactation [48]. The absence of WAP in human and the NMR agrees with the low levels of total protein in their milk relative to mice, rats, and rabbits [44, 49]. Its expression levels of 18 and 53 TPM were demonstrated in the mammary gland in experiments, while it never exceeded 3 TPM in other tissues [26].

The mouse gene *Wfdc16* (ENSMUSG00000070530) has orthologs only in murine rodents and some Laurasiatheria species. In particular, it was lost in the NMR, BMR, DMR, chinchilla, and all primates. *Wfdc16* is most actively expressed in the epididymis and pregnant uterus. The protein product is thought to be an inhibitor of peptidases. The rat has at least four WFDC-containing proteins; three of them, Wfdc8, Wfdc11, and Wfdc16, are expressed only in the epididymis [50]. Their expression depends on the levels of sex hormones [51]. The action of at least one of these proteins, Wfdc8, pertains to antimicrobial defense [50]. According to [27], its expression in the genital fat pad (120 RPKM) is 30 times higher than in organs not directly related to reproduction (4 RPKM in kidneys), and there is also significant expression (7 RPKM) in the testis. According to [26], one experiment revealed that its expression in the epididymis was 330 TPM, which is 20 times that in the kidney and other tissues not directly related to reproduction (but 21 TPM in the testis and 20 TPM in the uterus). The biological properties, primarily the tissue-specific expression of the identified genes, are detailed in Additional file 3. These results indicate that the genes identified by our program directly relate to reproduction and, according to Williams’ theory [23], can influence the lifespan.

Let us return to the discussion of predicted genes in general. Low levels of their expression are commonly observed in many organs, which can be potentially harmful. Certain age-related (e.g., oncological and neurodegenerative) diseases can progress slowly so as not to affect young organisms [52]. Accordingly, individuals of short-lived species largely do not survive to the age when the disease becomes a problem. This exemplifies the antagonistic pleiotropy, i.e., the opposite effects of the same gene during ontogeny [23].

On the other hand, the loss of certain genes might not be a factor of increased lifespan but rather may result from an increased lactation period and reproductive age. This indicates that a gene loss can accompany longevity without contributing to its cause.

According to the common point of view, rearrangements in the network of gene cis-regulatory elements are considered to be the main route for the evolutionary changes that lead to the phenotypic and physiological differences between species, particularly in vertebrates [53]. Unfortunately, this kind of genomic rearrangement is difficult to reveal by systematic screening using bioinformatic methods. The identification of cis-regulatory elements is a complex task, and mutations are not necessarily linked to the functioning of the corresponding gene. Clearly, gene losses do not exclude such evolutionary events as well.

The limitations of the method include a certain dependence on the results of the orthology inference method and some sensitive parameters, such as the vicinity size or the number of witnesses in the upper and lower species. Accordingly, we selected only genes of the reference species that were lost for all or most of these parameters.

## Conclusions

The developed method and its software allowed us to identify a short list of presumably lost genes (i.e., genes with disrupted orthology or local synteny) associated with a long lifespan in Euarchontoglires. The predicted lost genes largely demonstrate specific expressions in reproductive organs, which agrees with Williams’ hypothesis [23] concerning the reallocation of the physiological resources of the body between self**-**maintenance and reproduction (transition to К-strategy in the species evolution). The loss of some predicted vomeronasal and olfactory receptor genes in human and naked mole-rat conforms to their specific anatomical features. We suggest that the loss of certain genes in evolution is one of the essential determinants of lifespan. Overall, it is a likely driving force for many aspects of species evolution in vertebrates.

## Methods

**Fig. 4:**
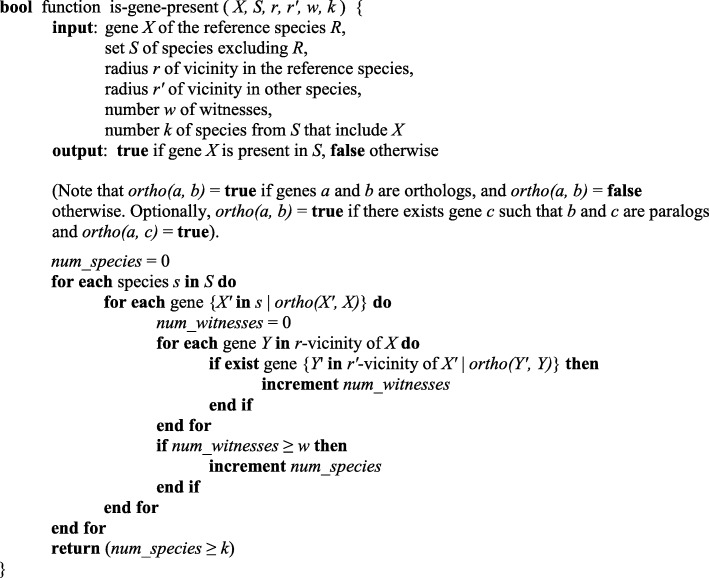
The pseudocode to test whether gene *X* is present in the set of species *S*

This section details the program-based realization of our main result as specified above in “*The method of in silico search for lost genes*”. Lost genes were searched using our program lossgainRSL [6]. It allows the user to identify the genes of a given reference species that are present or lost in several predefined sets of species in accordance with a given logical function (predicate). The elementary condition is “gene *X* is present in the set of species *S*”. It is tested by the function shown in Fig. 4.

Thus, gene *X* is present in the set *S* if it is present in at least *k* species from *S* (*k* is a set-specific parameter). The *presence* of gene *X* from the reference species in another species means that (*i*) the latter species includes the gene *X’* which is an ortholog of *X* and (*ii*) there are several other genes (“witnesses”) near gene *X* that have respective orthologs located near *X’* in that species (see Fig. 1). If either condition is not satisfied, the gene is considered *lost*. Here, the orthologs may be of any type (1-to-1, 1-to-many, many-to-many), and the program can optionally accept a gene *X’* that is not orthologous to *Х* but is a paralog of an ortholog of gene *Х*. Thus, synteny is taken into account in addition to orthology and paralogy; the consideration of synteny significantly advances the instrumentation for the identification of genes lost (or gained) in evolution. The program lossgainRSL does not rely on a certain orthology inference method and uses orthology data at the input.

The abovementioned predicate is a Boolean function composed of such conditions using connectives AND, OR, and NOT; it is described below.

The sets of species, predicate, number of witnesses, other synteny details such as the values of *r* and *r’* for each elementary condition, and orthology type are the program parameters. In particular, the gene order and direction in a synteny block can be the same (as shown in Fig. 1) or differ within the specified conditions. Here, we varied the *r* and *r’* values from 1 to 5 Mbp.

All protein-coding genes from the reference species are tried one by one. For each gene *X*, the algorithm verifies the predicate until the first match; then, the gene is added to the general list of identified genes; otherwise, it is skipped. The resulting list includes genes from the reference species that are present in some species and are missing in others following the predicate. Proper selection of species groups and elementary conditions included in the predicate allows the program to identify both lost and acquired genes. The program is somewhat sensitive to the quality of the genome assembly: short contigs containing one or a few genes as well as genes located at the contig boundary can be difficult to process. The method does not require chromosome-level assembly; it can process contigs of arbitrary length.

The current version 6.20 of lossgainRSL is primarily targeted at species and genomic data included in the Ensembl database [25], but it also provides for other orthology data as described in the program manual. The program uses three types of input data, that is, three tables, one of genes, one of orthologs, and one of paralogs for each relevant species. The table of paralogs is necessary if the selection of orthologous genes should be performed with the paralogs considered. A species from GenBank can also be added using the utility ‘addspecies’, which is also available at the program site. The lossgainRSL output is tab delimited and can be imported into Excel. This file contains all identified genes of the reference species that satisfy the predicate with the given parameters. The output can optionally contain the entire synteny block (i.e., including the witnesses) for each gene as well as their orthologs in other species.

**Fig. 5:**
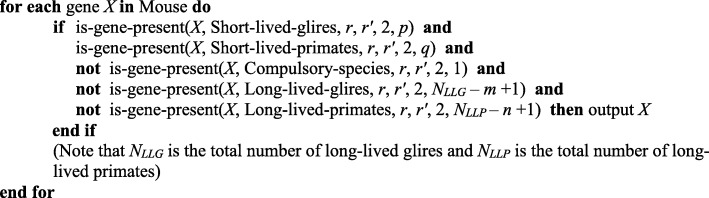
Dynamically generated pseudocode that yields the list of lost genes

The program was implemented in C++ as a command-line utility for Windows/Linux. It can perform parallel computing in the MPI environment and uses load balancing according to [54], which allows processing a considerable number of complete genomes within a reasonable time (data not shown). The configuration file is required to run the program; it is a text file composed of a number of sections, each starting with a section name in square brackets on a separate line (see Additional file 4). The crucial part of the configuration file is the section named [predicate], which specifies the Boolean function for the selection of genes in the reference species. The function can be of complex and branchy design; it is specified implicitly as a chain of tests for each gene using a programming language-like syntax. The program interprets this description, thus generating a dynamic code. The above results were obtained using the code in Fig. 5 invoking the function shown in Fig. 4.

Thus, this algorithm takes into account the two groups of Euarchontoglires species mentioned in the Results section. Each group includes the upper and lower fractions, which are tentative long- and short-lived species, respectively. These groups and fractions are detailed below. In addition, the NMR, human, and capuchin are considered compulsory species where gene *X* must be absent.

The corresponding configuration file is given in Additional file 4. It illustrates the potential of the language describing the predicates and was designed to reflect how the predicate is satisfied in detail. Specifically, all but the compulsory species will be shown in the output file. Only the Ensembl ID is given for orthologous genes in those species, but the configuration file can be easily changed to return more information, such as the names, coordinates, and annotations of these genes (as was done for the mouse genes). In addition, the identified gene and its orthologs can be supplemented by the whole syntenic block containing neighboring witnesses in each species. All 134 genes identified using this configuration file are listed in Additional file 2.

The program has been extensively parallelized and can operate on a supercomputer, which is essential if many complete genomes are considered or if synteny blocks consist of many neighboring genes. However, in the case of Ensembl v95 data and the program parameters listed in the Results section, the execution time did not exceed 10 min on a PC with a 6-core Xeon processor.

Here, the method for the identification of lost genes described in the first section of the Results and in this section was applied in the context of the species lifespan (longevity) as follows. Forty Euarchontoglires species falling into two groups were considered: the first included 16 rodents and lagomorphs, and the second, 24 primates (see Table 1). The upper and lower fractions of species were defined by the quantitative longevity index. Several indices are known: the longevity quotient (LQ), the maximum longevity residual (MLR), the maximum reported lifespan (MRLS), etc. Here, we used the AnAge build 14 database [18] and its index, referred to as LQ below. It is calculated using the formula MRLS/(4.88∙*m*^0.153^), where *m* is the adult body weight in grams as specified in the database. We have selected the species listed in Table 1 considering their representation in Ensembl and the quality of the genomes addressed in Additional file 5: Table S2, which does not always match a biologically justified selection. Among numerous publications on the relationship between MRLS and body weight and related problems, one should mention [21, 55, 56].

While compiling the two sets, that is, long- and short-lived species, we arranged species in Table 1 as follows: species were sorted by descending LQ *separately* for glires and primates, after which their upper fractions were joined and opposed to the joined lower fractions. Specifically, we included in the upper set three rodents with LQ > 130% and seven primates with LQ > 220% and accepted that the NMR, human and white-headed capuchin are compulsory species where the gene must be absent. The lower set consisted of eleven rodents and lagomorphs with LQ < 110% and ten primates with LQ < 190%. The mouse was a reference species.

We do not discuss the global biological problem of longevity because it combines many complex phenomena including resistance to cancer. The following opinion [21] illustrates this problem well: “Aging is a complex process affecting different species and individuals in different ways. Comparing genetic variation across species with their aging phenotypes will help to understand the molecular basis of aging and longevity. Although most studies on aging have so far focused on short-lived model organisms, recent comparisons of genomic, transcriptomic, and metabolomic data across lineages with different lifespans are unveiling molecular signatures associated with longevity.” We believe that the quote applies well to the proposed program.

The data on the genomes and gene expression were obtained from Ensembl v95 [25], Expression Atlas release 29 [26], and Mouse ENCODE [27].

## Supplementary information

**Additional file 1.** Additional information on some long-lived mammals.

**Additional file 2: Table S1.** Table of identified mouse genes lost in mammals with a long lifespan. Columns: A, Ensembl gene ID; B, postprocessing result, where 0 means that an upper species has indications of gene preservation; C–F, maximum values of *m*, *n*, *p*, *q* thresholds, respectively, for the mouse gene to be still identified; G–J, coordinates of the gene; K, L, the name and description of the gene; M–AV, orthologous genes in other species specified in the head row.

**Additional file 3:.** Additional information on some predicted loss genes.

**Additional file 4:.** The contents of the lossgainRSL program configuration file used to identify mouse genes lost in mammals with a long lifespan.

**Additional file 5: Table S2.** Data table of genomes in Ensembl v95. Columns: A, scientific name; B, common name; C, assembly name; D, accession and version; E, genome total length; F, total length of the assembly gaps; G, gaps percentage; H, total number of scaffolds; I, scaffold N50; J, total number of protein-coding genes in the Ensembl database; K, number of genes that have at least two neighboring genes; L, percentage of protein-coding genes having two or more neighbors.

## Data Availability

All data generated or analyzed during this study are included in this published article and its supplementary information files. The code of the software, the test example, and the documentation are available in the FigShare repository, 10.6084/m9.figshare.9173243.v2

## Abbreviations

BMR: Upper Galilee mountains blind mole rat
DMR: Damaraland mole-rat
LQ: longevity quotient
MRLS: maximum reported lifespan
NMR: naked mole-rat
RPKM: reads per kilobase per million mapped reads
TAD: topologically associated domain
TPM: transcripts per million
