# Supplementary material for: Screening for mouse genes lost in mammals with long lifespans
Source: BioData Min. 2019 Nov 9;12:20. doi: 10.1186/s13040-019-0208-x (PMC6842137; doi:10.1186/s13040-019-0208-x)
Supplement: Supplementary file 1 — Additional file 1. Additional information on some long-lived mammals. [file 13040_2019_208_MOESM1_ESM.doc]

# Additional File 1

**for the article “Screening for mouse genes lost in mammals with long lifespans” by**

**Rubanov LI, Zaraisky AG, Shilovsky GA, Seliverstov AV, Zverkov OA, and Lyubetsky VA**

Following our general hypothesis on the evolutionary significance of gene loss [1], we tried to identify lost genes in the mole rats and primates with a longer lifespans than could be expected from their body size: naked mole-rat, Upper Galilee mountains blind mole rat, Damaraland mole rat, white-headed capuchin, Sumatran orangutan, northern white-cheeked gibbon, bonobo, chimpanzee, Bolivian squirrel monkey (*Saimiri boliviensis*), and human. Species living under different conditions were analyzed together to reduce the habitat impact on the phenomenon studied. Specifically, the genes independently present and lost in at least two phylogenetic groups were selected. We consider 40 species belonging to the superorder Euarchontoglires.

Among these, let us note the species with a high lifespan. The naked mole-rat (NMR, *Heterocephalus glaber*) is an eusocial mammal with a very long lifespan for rodents, over 32 years, during which senile changes are scarcely observed and the death rate is extremely low [2, 3, 4]. On the other hand, the NMR displays the same types of cellular senescence found in a short-lived rodent [5]. The NMR is resistant to cancer as well as the Damaraland mole rat (DMR, *Fukomys damarensis*). Genome comparison of these mole rats and other species indicate common mechanisms of adaptation to oxygen deprivation as well as at high levels of ammonia and carbon dioxide; the comparison indicates the absence of at least one of the two main receptors for melatonin, a pineal hormone and circadian rhythm regulator [6]. The NMR and DMR also have a similar social structure. Although DMR has a lower lifespan, it is the closest relative of NMR (among the species considered). Accordingly, it is natural to expect that certain genes lost in the NMR and DMR had been lost in their common ancestor. The mole rats are excellent models for studying mammalian adaptation. The DMR is remarkable for the traits that make genomic and transcriptomic information on it useful for biomedical research, in particular, in cancer study [6]. The Upper Galilee mountains blind mole rat (BMR, *Spalax galili*) spends its entire life underground and challenges with multiple stressors such as darkness, hypoxia, hypercapnia, energetics and high pathogenicity [7]. The phylogenetic position of the BMR is much more distant from the NMR. Accordingly, the gene losses in the NMR and BMR can be considered independent evolutionary events. In general, longer lifespans are observed in apes (Hominoidea) and capuchins (Cebinae). The white-headed capuchin has a lifespan comparable to those of the gorilla and chimpanzee while its weight is lower [8]. These considerations, as well as the completeness of genome assemblies, were used to select the considered 40 species tabulated in the Methods section.

Previously, gene loss or acquisition largely was considered as a change in the number of gene paralogs [9]. Later, a gene loss in a species was proposed to be an important radical genomic change with a high potential for evolutionary adaptations [10, 11]. The significance of studying the role of gene loss in evolution, the “avenue for future research would be the role of gene loss during the evolution of long‐lived species,” is pointed out in [12] along with that of gene duplications. A classic example is the loss of the myosin heavy chain 16 (MYH16) gene in human (but not in other primates) possibly resulting from diet changes. It has relieved the dependence on powerful masticatory muscle; this event (~2.4 million years ago) increased the intracranial volume and brain size [13]. Other similar examples are the losses of the gene encoding CMP-N-acetylneuraminic acid hydroxylase underlying the resistance to certain pathogens [14] and caspase-12, a factor of resistance to severe sepsis, which occurred shortly before human migration from Africa [15]. The restructuring of a static set of genes in the regulatory network can cause an evolutionary change as well.

Analysis of the Hox family genes demonstrated that sequence alignment and synteny can provide conflicting orthology patterns [16]. Among other things, this is due to different evolutionary rates of genes and chromosome structures. Murine rodents show the highest rate of changes in chromosome structures among studied mammals; their chromosomes tend to accumulate protein-coding genes, specifically those associated with reproduction and pheromone recognition, especially in the regions of disrupted synteny [17]. In computer analysis, “chromosome structure” is considered in a narrow sense as a mutual arrangement of genes with no account of many other chromosome properties [18, 19].

**REFERENCES**

1. Korotkova DD, Lyubetsky VA, Ivanova AS, Rubanov LI, Seliverstov AV, Zverkov OA, Martynova NYu, Nesterenko AM, Tereshina MB, Peshkin L, Zaraisky AG. Bioinformatic screening of genes present only in well regenerating vertebrates reveals novel FGF and purinergic signaling modulator - c-Answer. *Cell Reports*. 2018;29(4):1027–40.
2. Buffenstein R. Negligible senescence in the longest living rodent, the naked mole-rat: insights from a successfully aging species. *J. Comp. Physiol. B.* 2008;178:439-45.
3. Lewis KN, Soifer I, Melamud E, Roy M, McIsaac RS, Hibbs M, Buffenstein R. Unraveling the message: insights into comparative genomics of the naked mole-rat. *Mamm. Genome*. 2016;27(7-8):259-78. doi:10.1007%2Fs00335-016-9648-5.
4. Ruby JG, Smith M, Buffenstein R. Naked mole-rat mortality rates defy gompertzian laws by not increasing with age. *eLife*. 2018;7: e31157. doi:10.7554/eLife.31157.
5. Zhao Y, Tyshkovskiy A, Muñoz-Espín D, Tian X, Serrano M, de Magalhães JP, Nevo E, Gladyshev VN, Seluanov A, Gorbunova V. Naked mole rats can undergo developmental, oncogene-induced and DNA damage-induced cellular senescence. *Proc Natl Acad Sci U S A*. 2018;115(8):1801-6. doi:10.1073/pnas.1721160115.
6. Fang X, Seim I, Huang Z, Gerashchenko MV, Xiong Z, Turanov AA, Zhu Y, Lobanov AV, Fan D, Yim SH, Yao X, Ma S, Yang L, Lee SG, Kim EB, Bronson RT, Šumbera R, Buffenstein R, Zhou X, Krogh A, Park TJ, Zhang G, Wang J, Gladyshev VN. Adaptations to a subterranean environment and longevity revealed by the analysis of mole rat genomes. *Cell Rep.* 2014;8(5):1354-64. doi:10.1016/j.celrep.2014.07.030.
7. Fang X, Nevo E, Han L, Levanon EY, Zhao J, Avivi A, Larkin D, Jiang X, Feranchuk S, Zhu Y, Fishman A, Feng Y, Sher N, Xiong Z, Hankeln T, Huang Z, Gorbunova V, Zhang L, Zhao W, Wildman DE, Xiong Y, Gudkov A, Zheng Q, Rechavi G, Liu S, Bazak L, Chen J, Knisbacher BA, Lu Y, Shams I, Gajda K, Farré M, Kim J, Lewin HA, Ma J, Band M, Bicker A, Kranz A, Mattheus T, Schmidt H, Seluanov A, Azpurua J, McGowen MR, Ben Jacob E, Li K, Peng S, Zhu X, Liao X, Li S, Krogh A, Zhou X, Brodsky L, Wang J. Genome-wide adaptive complexes to underground stresses in blind mole rats Spalax. *Nat Commun*. 2014;5:3966. doi:10.1038/ncomms4966. *Erratum in Nat Commun*. 2015;6:8051.
8. de Magalhães JP, Sedivy JM, Finch CE, Austad SN, Church GM. A proposal to sequence genomes of unique interest for research on aging. *J. Gerontol. A Biol. Sci. Med. Sci*. 2007;62:583-4.
9. Force A, Lynch M, Pickett FB, Amores A, Yan YL, Postlethwait J. Preservation of duplicate genes by complementary, degenerative mutations. *Genetics*. 1999;151(4):1531-45.
10. Cañestro C, Albalat R, Irimia M, Garcia-Fernàndez J. Impact of gene gains,losses and duplication modes on the origin and diversification of vertebrates. *Semin. Cell Dev. Biol*. 2013;24(2):83-94. doi:10.1016/j.semcdb.2012.12.008.
11. Albalat R, Cañestro C. Evolution by gene loss. *Nat. Rev. Genet*. 2016;17(7):379-91. doi:10.1038/nrg.2016.39.
12. Doherty A, de Magalhães JP. Has gene duplication impacted the evolution of Eutherian longevity? *Aging Cell*. 2016. 15:978-80. doi: 10.1111/acel.12503.
13. Stedman HH, Kozyak BW, Nelson A, Thesier DM, Su LT, Low DW, Bridges CR, Shrager JB, Minugh-Purvis N, Mitchell MA. Myosin gene mutation correlates with anatomical changes in the human lineage. *Nature*. 2004;428(6981):415-8. doi:10.1038/nature02358.
14. Chou HH, Hayakawa T, Diaz S, Krings M, Indriati E, Leakey M, Paabo S, Satta Y, Takahata N, Varki A. Inactivation of CMP-N-acetylneuraminic acid hydroxylase occurred prior to brain expansion during human evolution. *Proc. Natl. Acad. Sci. U S A*. 2002;99:11736-41.
15. Wang X, Grus WE, Zhang J. Gene losses during human origins. *PLoS Biol.* 2006;4(3):e52. doi:10.1371/journal.pbio.0040052.
16. Hueber SD, Rauch J, Djordjevic MA, Gunter H, Weiller GF, Frickey T. Analysis of central Hox protein types across bilaterian clades: on the diversification of central Hox proteins from an Antennapedia/Hox7-like protein. *Dev. Biol*. 2013;383(2):175-85. doi:10.1016/j.ydbio.2013.09.009.
17. Capilla L, Sánchez-Guillén RA, Farré M, Paytuví-Gallart A, Malinverni R, Ventura J, Larkin DM, Ruiz-Herrera A. Mammalian comparative genomics reveals genetic and epigenetic features associated with genome reshuffling in Rodentia. *Genome Biol. Evol.* 2016;8(12):3703-17. doi:10.1093/gbe/evw276.
18. Lyubetsky VA, Gershgorin RA, Seliverstov AV, Gorbunov KYu. Algorithms for reconstruction of chromosomal structures. *BMC Bioinformatics*. 2016;17:40. doi:10.1186/s12859-016-0878-z.
19. Lyubetsky VA, Gershgorin RA, Gorbunov KYu. Chromosome structures: reduction of certain problems with unequal gene content and gene paralogs to integer linear programming. *BMC Bioinformatics*. 2017;18:537. doi:10.1186/s12859-017-1944-x.
