# Supplementary material for: Screening for mouse genes lost in mammals with long lifespans
Source: BioData Min. 2019 Nov 9;12:20. doi: 10.1186/s13040-019-0208-x (PMC6842137; doi:10.1186/s13040-019-0208-x)
Supplement: Supplementary file 3 — Additional file 3:. Additional information on some predicted loss genes. [file 13040_2019_208_MOESM3_ESM.doc]

# Additional File 3

**for the article “Screening for mouse genes lost in mammals with long lifespans” by**

**Rubanov LI, Zaraisky AG, Shilovsky GA, Seliverstov AV, Zverkov OA, and Lyubetsky VA**

The mouse gene *Ttc41* (ENSMUSG00000044937) encodes the tetratricopeptide repeat domain 41 and corresponds to a pseudogene in human. At the same time, this gene was preserved in the orangutan. The gene is actively expressed in the mouse testis. According to [1], its expression in testis in seven experiments varies from 35 to 80 TPM while expression in other organs does not exceed 5 TPM. For the *Gm595* (ENSMUSG00000079606) gene expression in the adult testis (ranges from 35 to 100 TPM in eight experiments) is at least 100 times higher than in other organs.

For the *4930504O13Rik* gene (ENSMUSG00000052642), expression in testis is at least 100 times higher than in any sample unrelated to the reproductive system. According to [1], its expression level in the testis varied from 157 to 356 TPM but never exceeded 2 TPM in other tissues. According to [2], its expression in the adult testis (66.5 RPKM) is 6000 times higher than in any sample unrelated to reproductive system (maximum is 0.11 RPKM in the liver). The encoded protein is a member of the lymphocyte antigen-6 (Ly6)/urokinase-type plasminogen activator receptor (uPAR) superfamily of proteins [3].

For the *4931406B18Rik* gene (ENSMUSG00000013353), expression in adult testis is at least 10 times higher than in any sample unrelated to the reproductive system. The gene is not essential for male fertility [4]. According to [2], its expression in the adult testis (3.5 RPKM) is 700 times higher than in any sample unrelated to the reproductive system. According to [1], its expression in the testis varied from 10 to 21 TPM in six experiments but never exceeded 1 TPM in other tissues (excluding the 6-TPM expression in the embryonic medial nasal prominence).

For the gene *4921536K21Rik* (ENSMUSG00000020434), the expression in the testis is at least 20 times higher than in non-reproductive organs. According to [2], its expression in adult testis (23.8 RPKM) is 50 times higher than in any sample unrelated to the reproductive system. According to [1], its expression in the testis varied from 114 to 201 TPM in seven experiments but never exceeded 7 TPM in other tissues.

The *F830045P16Rik* gene (ENSMUSG00000043727) is predicted to localize in the plasma membrane; orthologous to human *SIRPB3P* (signal regulatory protein beta 3, pseudogene). Low expression observed in mice.

The genes *Cyct* (ENSMUSG00000056436) and *Ttc39d* (ENSMUSG00000046196) are expressed in the mouse testis (showing 100-fold expression differences in several experiments available in [1]) and are found in many apes, and the latter gene is also expressed in the DMR. However, both genes became pseudogenes in human. Mammalian testes express two types of cytochrome c, somatic cytochrome c, and testis-specific cytochrome c. The testis-specific one is the sole cytochrome c expressed in all postmeiotic male germ cells, including mature spermatozoa. Both cytochromes localize in the mitochondria in the same manner. The testis-specific cytochrome c-null mice testes undergo early atrophy equivalent to that which occurs during aging as a consequence of a reduction in oxidative phosphorylation [5].

The mouse gene *Cyp2ab1* (ENSMUSG00000022818) coding for a cytochrome P450 is most actively expressed in the placenta and heart [6], although its expression is also observed in the brain, liver, spleen, and skeletal muscle. In human, this gene corresponds to a pseudogene; however, the gene was preserved in the chimpanzee, bonobo, and gorilla. The gene has a lot of paralogs, 11 in the mouse and rat; 15 in the rabbit; but as low as 3 and 6 in the NMR and human, respectively. The family of cytochromes is too abundant (at least 57 in human and 102 in mouse) to reliably evaluate if the absence of *Cyp2ab1* and some of its paralogs is functional [6].

The *Hils1* gene (ENSMUSG00000038994) encoding for histone H1-like protein in spermatids 1 is mainly expressed in testis (50–100 times more actively than in any other organ). According to [1], its expression in the testis varied from 840 to 1660 TPM in seven experiments but never exceeded 14 TPM in other tissues.

The *Mrgprb8* gene (ENSMUSG00000050870) encoding for MAS-related GPR, member B8 has low expression in testis and no expression in other organs according to the six experiments available in [1].

**REFERENCES**

1. Papatheodorou I, Fonseca NA, Keays M, Tang YA, Barrera E, Bazant W, Burke M, Füllgrabe A, Fuentes AM, George N, Huerta L, Koskinen S, Mohammed S, Geniza M, Preece J, Jaiswal P, Jarnuczak AF, Huber W, Stegle O, Vizcaino JA, Brazma A, Petryszak R. Expression Atlas: gene and protein expression across multiple studies and organisms. *Nucleic acids research*. 2018;46:D246-51. doi:10.1093/nar/gkx1158.
2. Yue F, Cheng Y, Breschi A, Vierstra J, Wu W, Ryba T, Sandstrom R, Ma Z, Davis C, Pope BD, Shen Y, Pervouchine DD, Djebali S, Thurman B, Kaul R, Rynes E, Kirilusha A, Marinov GK, Williams BA, Trout D, Amrhein H, Fisher-Aylor K, Antoshechkin I, DeSalvo G, See LH, Fastuca M, Drenkow J, Zaleski C, Dobin A, Prieto P, Lagarde J, Bussotti G, Tanzer A, Denas O, Li K, Bender MA, Zhang M, Byron R, Groudine MT, McCleary D, Pham L, Ye Z, Kuan S, Edsall L, Wu YC, Rasmussen MD, Bansal MS, Keller CA, Morrissey CS, Mishra T, Jain D, Dogan N, Harris RS, Cayting P, Kawli T, Boyle AP, Euskirchen G, Kundaje A, Lin S, Lin Y, Jansen C, Malladi VS, Cline MS, Erickson DT, Kirkup VM, Learned K, Sloan CA, Rosenbloom KR, de Sousa BL, Beal K, Pignatelli M, Flicek P, Lian J, Kahveci T, Lee D, Kent WJ, Santos MR, Herrero J, Notredame C, Johnson A, Vong S, Lee K, Bates D, Neri F, Diege M, Canfield T, Sabo PJ, Wilken MS, Reh TA, Giste E, Shafer A, Kutyavin T, Haugen E, Dunn D, Reynolds AP, Neph S, Humbert R, Hansen RS, De Bruijn M, Selleri L, Rudensky A, Josefowicz S, Samstein R, Eichler EE, Orkin SH, Levasseur D, Papayannopoulou T, Chang KH, Skoultchi A, Gosh S, Disteche C, Treuting P, Wang Y, Weiss MJ, Blobel GA, Good PJ, Lowdon RF, Adams LB, Zhou XQ, Pazin MJ, Feingold EA, Wold B, Taylor J, Kellis M, Mortazavi A, Weissman SM, Stamatoyannopoulos J, Snyder MP, Guigo R, Gingeras TR, Gilbert DM, Hardison RC, Beer MA, Ren B, The mouse ENCODE Consortium. A comparative encyclopedia of DNA elements in the mouse genome. *Nature*. 2014 November 20. 515(7527):355-64. doi:10.1038/nature13992.
3. Loughner CL, Bruford EA, McAndrews MS, Delp EE, Swamynathan S, Swamynathan SK. Organization, evolution and functions of the human and mouse Ly6/uPAR family genes. *Human Genomics*. 2016;10:10. doi:10.1186/s40246-016-0074-2.
4. Miyata H, Castaneda JM, Fujihara Y, Yu Z, Archambeault DR, Isotani A, Kiyozumi D, Kriseman ML, Mashiko D, Matsumura T, Matzuk RM, Mori M, Noda T, Oji A, Okabe M, Prunskaite-Hyyrylainen R, Ramirez-Solis R, Satouh Y, Zhang Q, Ikawa M, Matzuk MM. Genome engineering uncovers 54 evolutionarily conserved and testis-enriched genes that are not required for male fertility in mice. *Proc. Natl. Acad. Sci. USA*. 2016;113(28):7704-10. doi:10.1073/pnas.1608458113.
5. Narisawa S, Hecht NB, Goldberg E, Boatright KM, Reed JC, Millán JL. Testis-specific cytochrome c-null mice produce functional sperm but undergo early testicular atrophy. *Molecular and Cellular Biology*. 2002;22(15): 5554-62. doi:10.1128/MCB.22.15.5554-5562.2002.
6. Renaud HJ, Cui JY, Khan M, Klaassen CD. Tissue distribution and gender-divergent expression of 78 cytochrome P450 mRNAs in mice. *Toxicol. Sci*. 2011;124(2):261-77. doi:10.1093/toxsci/kfr240.
