# Supplementary material for: Screening for mouse genes lost in mammals with long lifespans
Source: BioData Min. 2019 Nov 9;12:20. doi: 10.1186/s13040-019-0208-x (PMC6842137; doi:10.1186/s13040-019-0208-x)
Supplement: Supplementary file 4 — Additional file 4:. The contents of the lossgainRSL program configuration file used to identify mouse genes lost in mammals with a long lifespan. [file 13040_2019_208_MOESM4_ESM.doc]

# Additional File 4

**for the article “Screening for mouse genes lost in mammals with long lifespans” by**

**Rubanov LI, Zaraisky AG, Shilovsky GA, Seliverstov AV, Zverkov OA, and Lyubetsky VA**

The lossgainRSL program described in the main text requires a configuration file specifying the task to perform. This is a text (ASCII) file with unwrapped lines which are grouped into five sections each starting with a section name in square brackets on a separate line. Other lines can contain a number of tab-delimited fields. The lines that begin with semicolon are comments. This document provides the contents of the configuration file that we used to identify mouse genes lost in mammals with long lifespan. It illustrates the potential of the language describing the predicates and was designed to reflect the fulfillment of the gene selection conditions in more detail. The genes identified using this configuration file with the parameter values specified in the main text are listed in the Additional file 1.

; lossgainRSL configuration file

[mode]

; Program modes - single line, any order, case sensitive:

; Input: A = protein score matrix and table of genes

; C = cluster file(s) without gene information (use only Gene IDs)

; Q = cluster file(s) including gene information

; H = orthologous gene pairs and table of genes

; Synteny details: O = same order, D = same direction, M = mirror allowed

; Span calc: B = intercenter, S = intergenic, E = outer edges (default)

; Output: X = extract reference genes (otherwise, whole synteny block)

; I = separate each block with blank line (if not X)

; J = add line tail for sorting blocks

H E X

[data]

; In H mode, input files must be named as <species>.extension.

; O = table of orthologs, P = table of paralogs, H = united table

O orthologs\*.tsv

P paralogs\*.tsv

; In C mode, input file name(s) shall be provided in separate lines.

C clusters\gene-clusters.tsv

; In A mode, the matrix files must be named as <species>.extension

A scores\*.txt

; The table of proteins&genes must be named as <species>.extension

I genes\*.tsv

[fields]

; Data field names in the gene tables, one field per line with optional alias.

; Strict order: Protein Gene Contig Start End Strand [...]

Protein ID Protein

Gene ID Gene

Region Contig

Start

End

Strand +/-

Label Name

Description

[species]

; List of species or groups thereof, one per line.

; Group name begins with * and precedes the species of that group.

; First species is the reference one.

mus_musculus

*Long-lived

; rodents

heterocephalus_glaber_female

heterocephalus_glaber_male

; primates

homo_sapiens

cebus_capucinus

*Putative_long-lived

; rodents

nannospalax_galili

fukomys_damarensis

chinchilla_lanigera

; primates

nomascus_leucogenys

pan_troglodytes

saimiri_boliviensis_boliviensis

pan_paniscus

pongo_abelii

aotus_nancymaae

macaca_fascicularis

macaca_mulatta

*Medium-lived

; rodents

octodon_degus

; primates

gorilla_gorilla

callithrix_jacchus

microcebus_murinus

macaca_nemestrina

*Short-lived

; rodents

dipodomys_ordii

peromyscus_maniculatus_bairdii

cavia_porcellus

jaculus_jaculus

ictidomys_tridecemlineatus

microtus_ochrogaster

oryctolagus_cuniculus

cavia_aperea

mesocricetus_auratus

rattus_norvegicus

; primates

colobus_angolensis_palliatus

mandrillus_leucophaeus

chlorocebus_sabaeus

propithecus_coquereli

carlito_syrichta

rhinopithecus_roxellana

otolemur_garnettii

cercocebus_atys

papio_anubis

rhinopithecus_bieti

[predicate]

; A predicate to be computed for each gene of the reference species.

SET,2,%r,7 // WIT[NESS],RANGE,BBH/ORTH,HOLD1,HOLD2,EVAL

HEAD Mus musculus,Region,Start,End,Strand,Name,Description,Nannospalax galili,Fukomys damarensis,Chinchilla lanigera,Nomascus leucogenys,Pan troglodytes,Saimiri boliviensis,Pan paniscus,Pongo abelii,Aotus nancymaae,Macaca fascicularis,Macaca mulatta,Octodon degus,Gorilla gorilla,Callithrix jacchus,Microcebus murinus,Macaca nemestrina,Dipodomys ordii,Peromyscus maniculatus,Cavia porcellus,Jaculus jaculus,Ictidomys tridecemlineatus,Microtus ochrogaster,Oryctolagus cuniculus,Cavia aperea,Mesocricetus auratus,Rattus norvegicus,Colobus angolensis,Mandrillus leucophaeus,Chlorocebus sabaeus,Propithecus coquereli,Carlito syrichta,Rhinopithecus roxellana,Otolemur garnettii,Cercocebus atys,Papio anubis,Rhinopithecus bieti

ORTH 6

; Absent from compulsory long-lived species

IN *Long-lived NO

ADD mus_musculus Gene,Region,Start,End,Strand,Name,Description

; Present in not greater than %m long-lived rodents

BOR

IN nannospalax_galili

ADD nannospalax_galili Gene

IN fukomys_damarensis

ADD fukomys_damarensis Gene

IN chinchilla_lanigera

ADD chinchilla_lanigera Gene

EOR,%m NO

; Present in not greater than %n long-lived primates

BOR

IN nomascus_leucogenys

ADD nomascus_leucogenys Gene

IN pan_troglodytes

ADD pan_troglodytes Gene

IN saimiri_boliviensis_boliviensis

ADD saimiri_boliviensis_boliviensis Gene

IN pan_paniscus

ADD pan_paniscus Gene

IN pongo_abelii

ADD pongo_abelii Gene

IN aotus_nancymaae

ADD aotus_nancymaae Gene

IN macaca_fascicularis

ADD macaca_fascicularis Gene

IN macaca_mulatta

ADD macaca_mulatta Gene

EOR,%n NO

ORTH 7

; Check medium-lived species

IN octodon_degus

ADD octodon_degus Gene

IN gorilla_gorilla

ADD gorilla_gorilla Gene

IN callithrix_jacchus

ADD callithrix_jacchus Gene

IN microcebus_murinus

ADD microcebus_murinus Gene

IN macaca_nemestrina

ADD macaca_nemestrina Gene

; Present in at least %p short-lived rodents

BOR

IN dipodomys_ordii

ADD dipodomys_ordii Gene

IN peromyscus_maniculatus_bairdii

ADD peromyscus_maniculatus_bairdii Gene

IN cavia_porcellus

ADD cavia_porcellus Gene

IN jaculus_jaculus

ADD jaculus_jaculus Gene

IN ictidomys_tridecemlineatus

ADD ictidomys_tridecemlineatus Gene

IN microtus_ochrogaster

ADD microtus_ochrogaster Gene

IN oryctolagus_cuniculus

ADD oryctolagus_cuniculus Gene

IN cavia_aperea

ADD cavia_aperea Gene

IN mesocricetus_auratus

ADD mesocricetus_auratus Gene

IN rattus_norvegicus

ADD rattus_norvegicus Gene

EOR,%p -NO

; Present in at least %q short-lived primates

BOR

IN colobus_angolensis_palliatus

ADD colobus_angolensis_palliatus Gene

IN mandrillus_leucophaeus

ADD mandrillus_leucophaeus Gene

IN chlorocebus_sabaeus

ADD chlorocebus_sabaeus Gene

IN propithecus_coquereli

ADD propithecus_coquereli Gene

IN carlito_syrichta

ADD carlito_syrichta Gene

IN otolemur_garnettii

ADD otolemur_garnettii Gene

IN rhinopithecus_roxellana

ADD rhinopithecus_roxellana Gene

IN cercocebus_atys

ADD cercocebus_atys Gene

IN papio_anubis

ADD papio_anubis Gene

IN rhinopithecus_bieti

ADD rhinopithecus_bieti Gene

EOR,%q -NO
